# Inverse regulation of serum osteoprotegerin and tumor necrosis factor-related apoptosis-inducing ligand levels in patients with leg lesional vascular calcification: An observational study

**DOI:** 10.1097/MD.0000000000014489

**Published:** 2019-03-08

**Authors:** Ae Ran Moon, Yoonkyung Park, Jeong Hwan Chang, Sang Su Lee

**Affiliations:** a Department of Biomedical Science and Research Center for Proteinaceous Materials, Chosun University; b Division of Vascular Surgery, Department of General Surgery, Cheomdan Medical Center, Gwang-Ju; c Vascular and endovascular division, Department of Surgery, Pusan National University Yangsan Hospital, Yangsan, Gyeongsangnam-do, Republic of Korea.

**Keywords:** diabetes, OPG, peripheral arterial disease, peroneal artery, popliteal artery, TRAIL, vascular calcification

## Abstract

We hypothesized that circulating osteoprotegerin (OPG) and tumor necrosis factor-related apoptosis-inducing ligand (TRAIL) levels could be associated with vascular calcification, which is predominant in diabetes.

The study included 71 Korean participants (36 with diabetes and 35 without diabetes), who were sub-grouped according to the results of the ankle–brachial index (ABI) and/or X-ray computed tomography scan (CT scan). Serum OPG and TRAIL levels were assayed using the respective enzyme-linked immunosorbent assay kits. Statistical significance was analyzed using Student's *t* test between the 2 groups or analysis of variance (ANOVA) among the 4 groups.

Serum OPG was up-regulated in the participants with diabetes, with peripheral arterial disease (PAD), and/or with vascular calcification. TRAIL down-regulation was more strictly controlled than OPG up-regulation; it was significantly downregulated in the participants with PAD and vascular calcification, but not in the participants with diabetes. Serum OPG and TRAIL were regulated in the participants with femoral, popliteal, and peroneal artery calcification but not in the participants with aortic calcification.

OPG up-regulation and TRAIL down-regulation were found to be associated with leg lesional vascular calcification; therefore, the average OPG/TRAIL ratio was significantly increased by 3.2-fold in the leg lesional vascular calcification group.

## 1 Introduction

Approximately 65% of patients with diabetes have cardiovascular complications. Vascular calcification is prevalent in diabetes and is a major cause of morbidity and mortality.^[1]^ This phenomenon is classified into 4 types according to the calcified lesions and the process mode, based on a histoanatomical perspective.^[2]^ Except for passively regulated vascular calcification in soft tissues, which is a minor type of calcification caused by increased calcium/phosphate products, the other 3 types of vascular calcification are actively regulated.^[3]^ These actively regulated vascular calcifications are morphologically divided into intimal and medial calcifications. Intimal calcification is observed in atherosclerosis and is caused by the association of macrophage, lipid, and vascular smooth muscle cells (VSMCs) in the arterial intima.^[4]^ In arterial intima calcification, arterial stiffness is increased, and vascular compliance is reduced due to fibrosis and increased calcification-induced impedance. Medial calcification is caused by elastin fiber mineralization in the arterial tunica media and is observed in end-stage renal disease and diabetes mellitus (DM).^[5]^ Although the mechanism of vascular calcification in diabetes is not yet fully elucidated, medial calcifications in muscle-type femoral and peroneal arteries are generally observed.^[6–8]^ Several studies have reported that medial calcification is induced by expressional alterations of osteoclastogenesis-related genes in VSMCs and vascular endothelial cells (VECs).^[6,7,9]^ Receptor activators of nuclear factor kappa-B (NFκB) ligand (RANKL) and receptor activators of nuclear factor kappa-B (RANK) are representative proteins associated with osteoclastogenesis.^[10]^ During bone resorption, RANKL binds to RANK in the membrane of osteoclast progenitor cells to activate osteoclasts, which then move to the fractured regions in bone via chemotaxis. Advanced glycation end products (AGEs), which are produced in diabetes, bind to the receptor for AGEs (RAGE) to induce NFκB activation, upregulation of RANKL, downregulation of insulin-like growth factor 1 receptor (IGF1R), and increased vascular calcium accumulation.^[11–13]^ Cytokines are increased by activated NFκB and regulate the expression of osteoprotegerin (OPG) and RANKL.^[13]^ Furthermore, RANKL is increased by inflammation, oxidized low-density lipoproteins (oxLDL), AGE, and reactive oxygen species (ROS), which bind to RANK in VSMC membranes causing them to differentiate.

OPG is a soluble decoy receptor of RANKL, which prevents RANKL–RANK binding and bone resorption. In OPG-deficient mice, vascular calcification in the aorta and renal arteries, as well as osteoporosis, was observed.^[14]^ OPG has been suggested to be a marker and risk factor for atherosclerosis and cardiovascular disease development due to its upregulation in atherosclerosis and cardiovascular disease.^[15,16]^ Additionally, aortic and circulating OPG levels are increased in patients with diabetes.^[9]^ Administration of TRAIL (TNF-related apoptosis-inducing ligand), a ligand of OPG, reduces atherosclerotic lesions in apolipoprotein E (ApoE) −/− mice with diabetes and protects against diabetic vascular injury in rats.^[17–19]^ Although the vascular calcification mechanism in diabetes remains debatable, there is evidence to indicate that bone remodeling system could be related to vascular calcification and diabetes.^[16–24]^

Although previous reports have suggested that OPG deficiency is related to aortic vascular calcification and that circulating OPG is upregulated in diabetes and vascular disease, the regulation mechanism of OPG is still unclear.^[9,14–16]^ Therefore, in this study, we aimed to clarify the relationship between OPG and TRAIL levels in vascular calcification and diabetes. Considering that OPG deficiency is related to aortic vascular calcification and circulating OPG is upregulated in diabetes, we hypothesized that circulating OPG regulation is associated with vascular calcification lesions, which are predominantly identified in diabetes. Because TRAIL is a ligand of OPG, serum levels of TRAIL could be related to the regulation of serum OPG levels. To test these hypotheses, the serum levels of OPG and TRAIL in Korean patients with and without diabetes associated with or without vascular calcification were examined. As calcifications in the femoral, popliteal, and peroneal arteries are prevalent in diabetes, the participants with vascular calcification were categorized based on the lesions of calcification.

## 2 Materials and methods

### 2.1 Participants

A total of 71 participants were recruited between February 2016 and April 2017 from 3 clinical centers in Korea (Appendix 1). The participants included 35 patients with diabetes (32 with vascular calcification) and 36 non-diabetic participants (15 with vascular calcification). The study protocol was approved by the institutional of the review committee in Chosun University Hospital IRB No 2014-07-014, the institution of the review committee in Pusan national university Yan San Hospital IRB No 05-2016-178 of at each clinical center, and written informed consent was obtained from all participants.

### 2.2 Ankle–brachial index

Systolic blood pressure was measured 3 times in each participant's arm (brachial artery) and ankle (posterior tibial artery) after 5 minutes of rest in the supine position. The ankle-brachial index (ABI) was calculated by dividing the mean of the brachial systolic blood pressure by the respective mean ankle systolic blood pressure.

### 2.3 X-ray computed tomography scan

Vascular calcification was investigated based on the results of a computed tomography (CT) scan using a 128-section dual-source CT system (SOMATOM Definition Flash, Siemens, Erlangen, Germany). Participants were scanned from the T12 upper intervertebral space to the distal toe following the intravenous injection of 1 to 2 mL/kg of iodinated contrast medium (Iopamidol; Pamiray-370, Dongkook Pharmaceutical, Seoul, Korea) at 0.5 mL/s. Obtained CT images were reconstructed using the filtered back projection algorithms, where the section thickness and section increment were set to 0.625 mm. All axial image data were exported to the picture archiving and communication system (PACS, Maroview; version 5.4.10.52, Marotech, Seoul, Korea). Vascular calcification was identified after comparison with the CT image of a non-calcified reference. Calcified lesions were divided into 3 zones: zones 1, 2, and 3 included the aortic and iliac arteries, femoral artery, and popliteal and peroneal arteries, respectively.

### 2.4 Enzyme-linked immunosorbent assay

The serum OPG assay was performed using a DuoSet enzyme-linked immunosorbent assay (ELISA) development system (DY805, R&D Systems, MN), with a human OPG capture antibody. Serum TRAIL levels were measured using Quantikine ELISA Human TRAIL/TNFSF10 immunoassay (DTRL00, R&D Systems, MN), in which the primary antibody-coated plate was provided. Assays were performed using 100 μL of serum, according to the respective protocols included in the kit. For the OPG assay, 96-well plates were coated with the provided capture antibody and incubated overnight. One-hundred microliters of serum from each participant was then added to both the OPG-coated 96-well plate and TRAIL-coated 96-well plate. After a 2-hour incubation at 18 to 20°C, each plate was washed 3 times with the indicated wash buffer in the respective protocol, before adding the detection antibody. After an additional 2-hour incubation at 18 to 20°C, plates were washed, and streptavidin-HRP (horseradish peroxidase) solution was added for color development. Optical density was measured using a microplate reader, Infinite M200 (Tecan, Männedorf, Switzerland), following sequential addition of substrate solution and stop solution. When the measured optical density was over 1.2, the assay was performed again using appropriately diluted serum with the indicated diluent in the kit. Serum OPG and TRAIL levels were obtained by calculation using the respective standard curve obtained using serially diluted OPG and TRAIL solutions provided in the kits.

### 2.5 Statistical analysis

Statistical significance was identified using the 2-tailed Student's *t* test to test for differences between 2 groups, or one-way analysis of variance (ANOVA) to test for differences between 4 groups. The Microsoft Excel 16.10 program was used and statistical significance was accepted when the *P* value was below .1.

## 3 Results

### 3.1 Serum OPG/TRAIL ratios were upregulated in diabetic participants with a low ABI result

**Figure 1:**
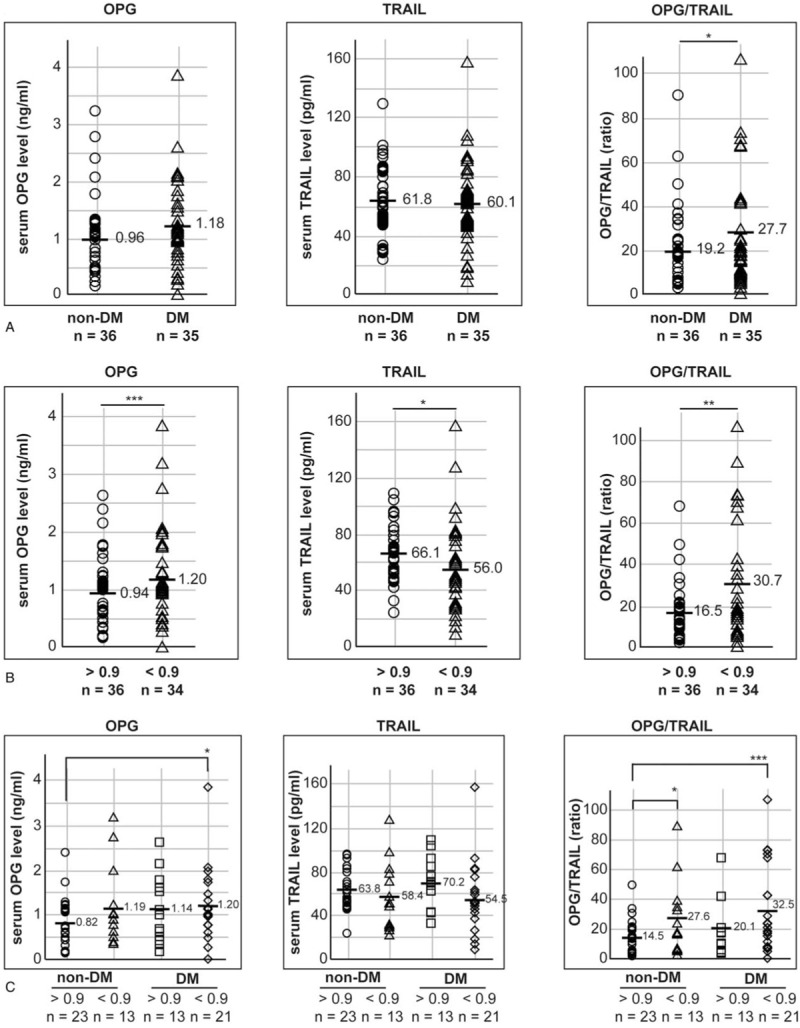
Serum OPG and TRAIL levels were regulated in participants with a low ABI. (A) Seventy-one participants were grouped as participants without diabetes (“non-DM”) and participants with diabetes (“DM”). Individual serum levels are marked as an open circle (○) and an open triangle (Δ) in the “non-DM” and “DM” groups, respectively. (B) Seventy participants were grouped based on the ABI result: 36 participants in the “ABI above 0.9” and 34 in the “ABI below 0.9” group. Individual serum levels are marked as an open circle (○) and an open triangle (Δ) in “ABI above 0.9” and “ABI below 0.9” groups, respectively. “n” indicates the number of participants. (C) Seventy participants were categorized based on ABI result and diabetes; the participants without diabetes and with a normal range ABI were grouped as a reference. Individual serum levels are marked as an open circle (○), open triangle (Δ), open rectangle (□), and an open diamond (◊) in “non-DM and ABI above 0.9”, “non-DM and ABI below 0.9”, “DM and ABI above 0.9”, and “DM and ABI below 0.9” groups, respectively. The average serum levels are marked as a bar (−) and the respective value. Statistical significance was identified by a 2-tailed Student's *t* test: ∗ and ∗∗ indicate *P* < .1 and *P* < .05, respectively. ABI = ankle–brachial index, DM = diabetes mellitus, OPG = osteoprotegerin, TRAIL = tumor necrosis factor-related apoptosis-inducing ligand.

To identify whether serum OPG and TRAIL levels were regulated in the participants with diabetes, serum OPG and TRAIL levels were compared between 35 patients with diabetes (the “DM” group) and 36 participants without diabetes (the “non-DM” group). Serum OPG levels tended to be increased in the “DM” group; however, the upregulation was not statistically significant, based on the 2-tailed Student's *t* test (*P* = .2; Fig. 1A). Furthermore, serum TRAIL levels were not regulated in the “DM” group. Nevertheless, a significant increase in the OPG/TRAIL ratio was observed in the “DM” group (*P* = .1), using the 2-tailed Student's *t* test.

A previous report indicated that OPG is not related to only diabetes but also to peripheral arterial disease (PAD), which is a well-known complication in diabetes; they investigated whether serum OPG and TRAIL levels could be regulated in the patients with PAD.^[25]^ To investigate whether OPG is regulated in PAD, the participants were categorized according to their ABI results, which is a well-known method for the diagnosis of PAD.^[26]^ Because the reference range of ABI is between 0.9 and 1.3, the participants were grouped into 2 groups: the “ABI below 0.9” (n = 36) and “ABI above 0.9” (n = 34) groups.^[26,27]^ One participant showing an ABI result of 1.33 was included into the “ABI above 0.9” group, and another participant who did not have an ABI result was excluded from this categorization. As seen in Figure 1B, serum OPG and TRAIL levels were up-regulated and down-regulated in the “ABI below 0.9” group (*P* < .01 and .1), respectively. Because of the OPG up-regulation and TRAIL down-regulation, the OPG/TRAIL ratio was significantly increased in the “ABI below 0.9” group (*P* = .01 with the 2-tailed Student's *t* test).

Although PAD is a well-known complication in diabetes, the possibility of a false-negative diagnosis of PAD has been reported in patients with diabetes when diagnosed with the ABI result.^[28]^ In the present study, serum OPG and TRAIL levels were regulated or tended to be regulated in PAD and diabetes, respectively (Fig. 1A and B). To investigate whether the serum OPG and/or TRAIL were regulated in diabetes and/or in diabetes with PAD, the participants were re-categorized into 4 groups based on the presence of diabetes and the ABI result: “non-DM and ABI above 0.9” (n = 23), “non-DM and ABI below 0.9” (n = 13), “DM and ABI above 0.9” (n = 13), and “DM and ABI below 0.9” groups (n = 21) (Fig. 1C).^[28]^ Although serum OPG levels were increased in the “ABI below 0.9” group (Fig. 1B), the statistically significant regulation of OPG was identified only in the “DM and ABI below 0.9” group and not in the “non-DM and ABI below 0.9” group. Serum TRAIL levels were decreased in the “ABI below 0.9” group (Fig. 1B); however, this was not statistically significant in “non-DM and ABI below 0.9” and the “DM and ABI below 0.9” groups because the levels were widely dispersed in each group (Fig. 1C). Nevertheless, the OPG/TRAIL ratio in the “DM and ABI below 0.9” group were significantly up-regulated (*P* = .01) due to the tendency for TRAIL down-regulation in the “DM and ABI below 0.9” group. The average OPG/TRAIL ratio in the “DM and ABI below 0.9” group (n = 21) was increased by 2.2-fold compared with that in the “non-DM and ABI above 0.9” group (n = 23). Because the OPG and TRAIL levels were widely dispersed within the groups, the median and average values are compared in Table 1.

**Table 1:**
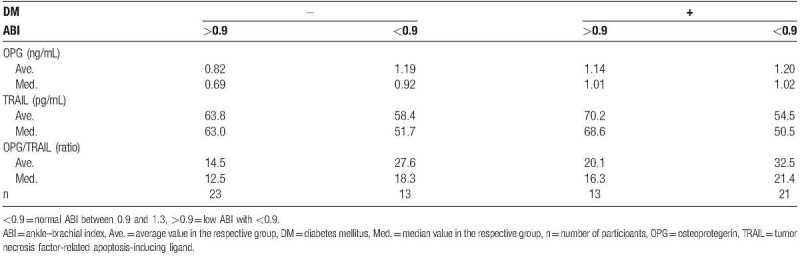
Comparison of the average and median OPG and TRAIL levels among the groups based on the diabetes and ABI result.

### 3.2 Serum OPG and TRAIL levels were significantly regulated in the vascular calcified participants showing a low ABI result

**Figure 2:**
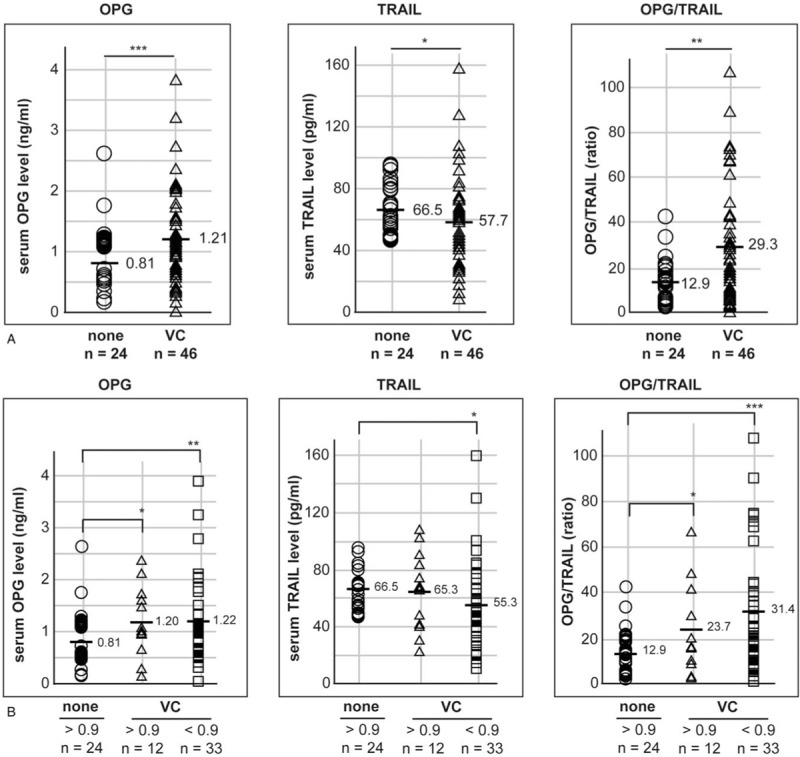
The regulation of serum OPG and TRAIL levels is related to the ABI result and vascular calcification. (A) Seventy participants were grouped based on the presence of vascular calcification into the “none” and “VC” groups. Individual serum levels are shown as an open circle (○) and an open triangle (Δ) in the “none” and “VC” groups, respectively. (B) Seventy participants were grouped based on the presence of vascular calcification and ABI result; the participants without calcified arteries and with a normal range ABI were grouped as a reference. Individual serum levels are shown as an open circle (○), open triangle (Δ), and an open rectangle (□), in “ABI above 0.9 and none”, “ABI above 0.9 and VC”, and “ABI below 0.9 and VC” groups, respectively. OPG/TRAIL ratios were calculated using individual serum OPG and TRAIL levels. “n” indicates the number of participants. The average serum levels and ratio are marked as a bar (−) and the respective value. Statistical significance was identified using Student's *t* test: ∗, ∗∗, and ∗∗∗ indicate *P* < .1, *P* < .05, and *P* < .01, respectively. ABI = ankle–brachial index, DM = diabetes mellitus, OPG = osteoprotegerin, TRAIL = tumor necrosis factor-related apoptosis-inducing ligand, VC = vascular calcification.

Vascular calcification is a major cause of low ABI (below 0.9).^[25,27,28]^ Therefore, we investigated whether serum OPG and TRAIL levels were regulated in PAD and/or in vascular calcification. The 70 participants were categorized according to the presence of vascular calcification as determined by the CT scan results. One participant was excluded due to no CT scan result (Fig. 2A). Serum OPG and TRAIL levels were inversely regulated, thus the average OPG/TRAIL ratio was higher by 2.2-fold in the “VC” (vascular calcification) group (n = 46) than in the “none” (no vascular calcification) group (n = 24).

Participants were re-categorized based on the ABI and CT scan results. Two participants were excluded; one did not have a CT scan result and the other did not have an ABI result. As no participants in the “none” group had an ABI below 0.9, the participants were divided into 3 groups: “ABI above 0.9 and none” (n = 24), “ABI above 0.9 and VC” (n = 12), and “ABI below 0.9 and VC” (n = 33). The regulation of serum OPG levels was more significant in the “ABI below 0.9 and VC” group (n = 33) than in the “ABI above 0.9 and VC” group (n = 12) (Fig. 2B). Although serum TRAIL levels in the “VC” group (n = 46) were down-regulated, as shown in Figure 2A, TRAIL regulation was only identified in the “ABI below 0.9 and VC” group (n = 33), and not in the “ABI above 0.9 and VC” group (n = 12). The average OPG/TRAIL ratio was increased by 2.4-fold and 1.8-fold in the “ABI below 0.9 and VC” and “ABI above 0.9 and VC” groups, respectively. Because serum OPG and TRAIL levels of the participants were widely distributed, the average and median values were compared, as shown in Table 2.

**Table 2:**
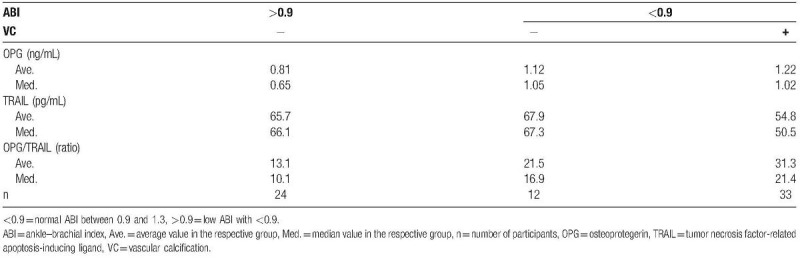
Comparison of the average and median OPG and TRAIL levels among the groups based on the ABI and CT scan results.

### 3.3 Serum OPG was up-regulated in diabetes with vascular calcification

**Figure 3:**
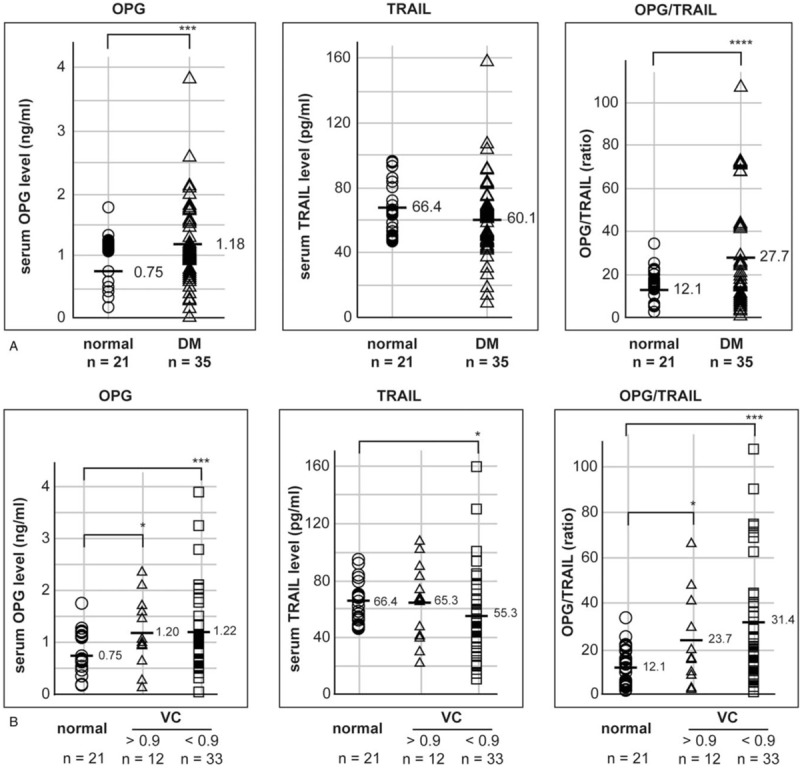
OPG up-regulation in participants with diabetes was identified by the re-establishment of a reference group. The reference group was re-established to exclude participants with a low ABI and vascular calcification, termed the “normal” group. (A) Seventy-one participants were grouped based on the presence of diabetes (n = 21 in the “normal group” and n = 35 in the “DM” group). Individual serum levels are marked as an open circle (○) and an open triangle (Δ) in the “normal” and “DM” groups, respectively. (B) Seventy participants were grouped based on the presence of vascular calcification and ABI result. Individual serum levels are shown as an open circle (○), open triangle (Δ), and an open rectangle (□), in the “ABI above 0.9 and none”, “ABI above 0.9 and VC”, and “ABI below 0.9 and VC” groups, respectively. Statistical significance was identified using Student's *t* test: ∗∗∗ and ∗∗∗∗ indicate *P* < .01 and *P* < .001, respectively. ABI = ankle–brachial index, DM = diabetes mellitus, OPG = osteoprotegerin, VC = vascular calcification.

Although serum OPG and TRAIL levels appeared to be regulated in vascular diseases but not in diabetes (Figs. 1 and 2), it is hard to exclude the possibility of a relationship between serum OPG and TRAIL levels and diabetes because serum OPG was significantly regulated in the “ABI below 0.9 and DM” group (Fig. 1). As some of the participants in the “non-DM” group (n = 36) belonged to the “ABI below 0.9” (n = 34) and/or “VC” (n = 46) groups, the regulation of serum OPG in diabetes was re-analyzed after the reference group was re-established to exclude the participants in the “ABI below 0.9” and/or “VC” groups from the “non-DM” group. This group was named the “normal” group (n = 21) (Fig. 3A). A up-regulation in the serum OPG and OPG/TRAIL ratio in the participants with diabetes was identified when comparing the “normal” (n = 21) and “DM” (n = 35) groups (*P* = .01 and *P* = .001, respectively); however, serum TRAIL levels were not regulated. Although serum OPG was up-regulated in the “DM” group, 32 of the 35 participants in the “DM” group also belonged to the “VC” group (n = 46). Therefore, whether serum OPG is regulated in diabetes without vascular calcification was not clear in this study.

In Figure 2B, the reference group, “ABI above 0.9 and none” (n = 24), included 3 participants with diabetes. Because serum OPG levels were up-regulated in the “DM” group (n = 35), the regulation of OPG and TRAIL levels were re-analyzed using a re-established reference group, in which the 3 participants with diabetes were excluded (Fig. 3B). The statistical significance between the “normal” (n = 21) and “ABI below 0.9 and VC” (n = 33) was increased. From these results, it is clear that serum OPG and TRAIL levels are regulated in patients with vascular calcification; however, the result remains unclear in diabetes.

### 3.4 Regulation of serum OPG and TRAIL levels were associated with vascular calcification

**Figure 4:**
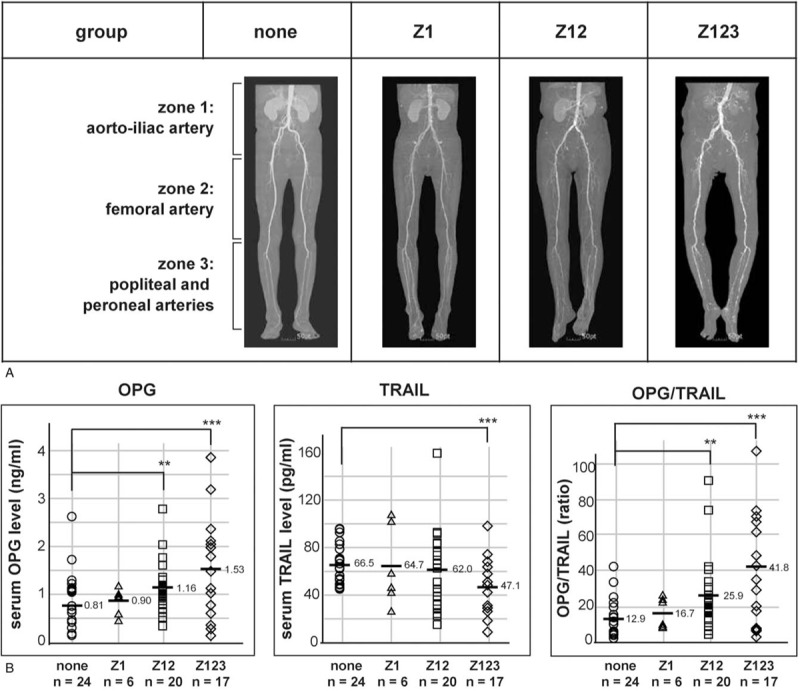
OPG and TRAIL levels were regulated in the participants with leg vascular calcification. Sixty-seven participants with vascular calcification were grouped based on the calcified artery. (A) The representative results of the CT scan corresponding to each group is shown: “Z 1” means “zone 1” (aortic and iliac arteries calcified); “Z 12” means “zones 1 and 2” (aortic, iliac, and femoral arteries calcified); “Z 123” means “zones 1, 2, and 3” (popliteal, peroneal, aortic, iliac, and femoral arteries calcified). (B) Individual serum levels are shown as an open circle (○), open triangle (Δ), open rectangle (□), and an open diamond (◊) in the “none”, “zone 1”, “zones 1 and 2”, and “zones 1, 2, and 3” groups, respectively. “n” indicates the number of participants. Average serum levels and the ratio are marked as a bar (−) and the respective value. Statistical significances between 2 groups or among 4 groups were identified by a 2-tailed Student's *t* test or single-factor ANOVA (*P* < .05), respectively: ∗, ∗∗, and ∗∗∗ indicate *P* < .1, *P* < .05, and *P* < .01, respectively. CT = computed tomography, OPG = osteoprotegerin, TRAIL = tumor necrosis factor-related apoptosis-inducing ligand.

Since vascular calcification is a major risk factor in diabetes and critical calcified lesions in diabetes are below the knee (BTK), the calcified artery lesions were divided into 3 zones: zone 1 included the aortic and iliac arteries; zone 2 included the femoral artery; and zone 3 included the popliteal and peroneal arteries (Fig. 4A).

The calcified artery lesions were dispersed in each participant, thus, they were grouped into 4 categories: the “none” group (n = 24) showed no calcified arteries; the “zone 1” group (n = 6) included participants with calcified arteries in zone 1; the “zones 1 and 2” group (n = 20) included participants with calcified arteries in zones 1 and 2; and the “zones 1, 2, and 3” group (n = 17) included participants, with calcified arteries in zones 1, 2, and 3. Three of the 70 participants were excluded: 1 had a calcified artery only in zone 2 and 2 had calcified arteries in zones 1 and 3.

Serum OPG levels were up-regulated in the “zones 1 and 2” (n = 20) and “zones 1, 2, and 3” (n = 17) groups, but not in the “zone 1” (n = 6) group (Fig. 4B). Serum TRAIL levels were significantly down-regulated in the “zones 1, 2, and 3” group. Although the number of the participants in the “zone 1” (n = 6) group was comparatively small, statistical significance was identified by comparing the OPG and TRAIL levels among the 4 groups (Fig. 4B; *P* = .015 and *P* = .1, respectively; One-way ANOVA). Especially, the up-regulation of OPG and down-regulation of TRAIL in the “zones 1, 2, and 3” group were remarkable. Serum OPG and TRAIL levels in the “zones 1, 2, and 3” group were increased to 190% and decreased to 70% of ‘none” group, respectively. Although *P* values in the analysis of TRAIL serum levels were at most .1 from the results of Figures 1–3, the *P* value between “none” and “zone 1, 2, and 3” groups was .005 (Fig. 4B). The average OPG/TRAIL ratio was significantly increased by 2-fold the “zones 1 and 2” group and by 3.2-fold in the “zones 1, 2, and 3” group. Because the numbers of participants in each group varied, the average and median values were compared, as shown in Table 3.

**Table 3:**
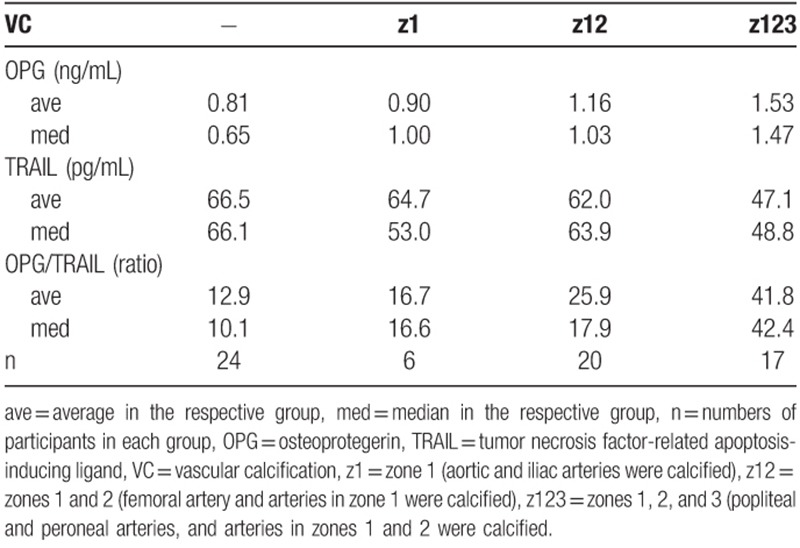
Comparison of the average and median OPG and TRAIL levels in each group based on the vascular calcified lesion.

## 4 Discussion

We examined the serum OPG and TRAIL levels in 71 study participants, including participants with diabetes, PAD, and/or with vascular calcification, to identify the relationship between serum OPG and TRAIL levels and diabetes, PAD, and/or vascular calcification. According to the study findings, serum OPG levels were significantly increased in participants with PAD (the “ABI below 0.9” group) or with vascular calcification (the “VC” group). An up-regulation in serum OPG was identified in diabetic participants (the “DM” group) when the reference group did not include participants with PAD or vascular calcification. Although serum OPG was up-regulated in the “DM” group, 91% of participants in the “DM” also belonged to the “VC” group; therefore, whether serum OPG levels were regulated in vascular calcification or in diabetes was not clear. Additionally, serum TRAIL was regulated in participants with vascular calcification and a low ABI result, but not in those with diabetes. Although TRAIL levels tended to be decreased in the “DM” group, the statistical significance was low (*P* = .2). This down-regulation of TRAIL may have resulted from the inclusion of 35% of the “DM” group participants belonging to the “ABI below 0.9 and VC” group.

According to previous reports, OPG is indicated to be associated with ongoing vascular disease, which is common in diabetes, and vascular calcification in the greater arteries has been observed in OPG-deficient mice.^[14,16]^ Additionally, TRAIL has been associated with diabetes in previous reports.^[23,24]^ Therefore, we hypothesized that serum OPG and TRAIL levels are differentially regulated according to the calcified lesion. We observed a significant regulation of OPG and TRAIL in participants with BTK vascular calcification (the “zones 1, 2, and 3” group), which is the most prevalent and critical calcified lesion in diabetes. It should be considered that 13 of the 17 (76%) participants in the “zones 1, 2, 3” group and 14 of the 20 (70%) participants in the “zones 1 and 2” group also belonged to the “ABI below 0.9” group, which could be the reason for the observed regulation of the OPG and TRAIL levels in the “ABI below 0.9 and VC” group. Interestingly, OPG and TRAIL were not modulated in the “zone 1” group. It has been previously reported that intima calcification and medial calcification are observed in atherosclerosis and diabetes, respectively, which suggested that the regulation of OPG and TRAIL levels are associated with medial calcification, but not intimal calcification.^[4,5]^

Serum TRAIL levels are more tightly regulated than serum OPG level; the regulation of serum TRAIL levels was identified only in the “ABI below 0.9 and VC” group and not in the “ABI below 0.9 and none” group (Fig. 2), as well as being significantly down-regulated in the “zones 1, 2, and 3” group (Fig. 4). As TRAIL and RANKL are competitive ligands of OPG, TRAIL down-regulation could increase OPG-RANKL binding. Therefore, RANKL-RANK binding could be decreased by OPG up-regulation and TRAIL down-regulation, inhibiting bone resorption. Because TRAIL was significantly regulated only in BTK vascular calcification (“zones 1, 2, and 3”) and OPG regulation was not identified in aortic calcification (“zone 1”), the inhibition of RANKL-RANK binding would be significant only in BTK vascular calcification. It could be suggested that the RANKL-OPG-TRAIL relationship is involved in the calcification mechanism in BTK arteries, but not in the aortic artery. It could also be hypothesized that bone resorption is associated with medial calcification but not intimal calcification.

In this context, it is possible that the increased OPG/TRAIL ratio could be an index to predict BTK calcification in diabetes. Although we analyzed serum RANKL levels in the participants in this study, there was no evidence to suggest a relationship between serum RANKL and vascular calcification or ABI. As calcification in the popliteal and peroneal arteries is a critical complication in diabetes, and CT scanning is the only method for identification of vascular calcification, these results are important in the diagnosis and management for diabetes and vascular disease. Further studies to determine whether the serum OPG and TRAIL levels of patients with diabetes could be indicators for the progression of diabetes or if the progression of diabetes could be controlled by regulating serum OPG and TRAIL levels are necessary.

All the participants with BTK calcification had calcified arteries from the aorta to the peroneal artery, except for 1 participant, who did not show the femoral and popliteal calcification (zone 2). Therefore, in this study, it could not be investigated whether the serum OPG and TRAIL levels would be regulated in the participants with vascular calcification depending on the localization and/or areas of the calcified artery. As the serum OPG and TRAIL levels are specifically regulated in the participants with BTK calcification, it is necessary to determine whether these regulations depend on the localization or area of the calcified artery, regardless of the presence or absence of diabetes. Further research should aim to determine whether TRAIL could function as an inhibitor of BTK vascular calcification because TRAIL was found to be specifically regulated in the participants with BTK calcification, but not in those with diabetes.

## 5 Conclusion

Serum OPG levels were significantly up-regulated in participants with vascular calcification. A down-regulation in TRAIL levels was identified in participants with PAD and/or with vascular calcification, especially with BTK vascular calcification. Although an up-regulation of serum OPG and down-regulation of TRAIL levels was observed in participants with calcified popliteal and peroneal arteries, it remains unclear whether the regulation of OPG and TRAIL is related to the location/area of the calcified artery.

## Author contributions

**Conceptualization:** Ae Ran Moon, Jeong Hwan Chang.

**Funding acquisition:** Ae Ran Moon, Yoonkyung Park, Jeong Hwan Chang, Sang Su Lee.

**Project administration:** Jeong Hwan Chang.

**Resources:** Yoonkyung Park.

**Supervision:** Jeong Hwan Chang, Sang Su Lee.

**Writing – original draft:** Ae Ran Moon.

**Writing – review & editing:** Sang Su Lee.

Sang Su Lee orcid: 0000-0003-0648-976X.

## References

[1] Sykes MT Godsey JB Vascular evaluation of the problem diabetic foot. Clin Podiatr Med Surg 1998;15:49–83. 9463768

[2] Fuery MA Liang L Kaplan FS Vascular ossification: pathology, mechanisms, and clinical implications. Bone 2017;S8756-3282:30232–6. 10.1016/j.bone.2017.07.006 28688892

[3] Abedin M Tintut Y Demer LL Vascular calcification: mechanisms and clinical ramifications. Arterioscler Thromb Vasc Biol 2004;24:1161–70. 15155384 10.1161/01.ATV.0000133194.94939.42

[4] Pugliese G Iacobini C Blasetti Fantauzzi C The dark and bright side of atherosclerotic calcification. Atherosclerosis 2015;238:220–30. 25528431 10.1016/j.atherosclerosis.2014.12.011

[5] Ho CY Shanahan CM Medial arterial calcification: an overlooked player in peripheral arterial disease. Arterioscler Thromb Vasc Biol 2016;36:1475–82. 27312224 10.1161/ATVBAHA.116.306717

[6] Collin-Osdoby P Regulation of vascular calcification by osteoclast regulatory factors RANKL and osteoprotegerin. Circ Res 2004;95:1046–57. 15564564 10.1161/01.RES.0000149165.99974.12

[7] Evrard S Delanaye P Kamel S Vascular calcification: from pathophysiology to biomarkers. Clin Chim Acta 2015;438:401–14. 25236333 10.1016/j.cca.2014.08.034

[8] Dawson S Lawrie A From bones to blood pressure, developing novel biologic approaches targeting the osteoprotegerin pathway for pulmonary vascular disease. Pharmacol Ther 2017;169:78–82. 27373854 10.1016/j.pharmthera.2016.06.017 PMC5243145

[9] Towler DA Shao JS Cheng SL Osteogenic regulation of vascular calcification. Ann N Y Acad Sci 2006;1068:327–33. 16831933 10.1196/annals.1346.036

[10] Hofbauer LC Heufelder AE Role of receptor activator of nuclear factor-kappaB ligand and osteoprotegerin in bone cell biology. J Mol Med (Berl) 2001;79:243–53. 11485016 10.1007/s001090100226

[11] Secchiero P Corallini F Pandolfi A An increased osteoprotegerin serum release characterizes the early onset of diabetes mellitus and may contribute to endothelial cell dysfunction. Am J Pathol 2006;169:2236–44. 17148684 10.2353/ajpath.2006.060398 PMC1762477

[12] Tseng W Graham LS Geng Y PKA-induced receptor activator of NF-kappaB ligand (RANKL) expression in vascular cells mediates osteoclastogenesis but not matrix calcification. J Biol Chem 2010;285:29925–31. 20663885 10.1074/jbc.M110.117366 PMC2943298

[13] Ndip A Wilkinson FL Jude EB RANKL-OPG and RAGE modulation in vascular calcification and diabetes: novel targets for therapy. Diabetologia 2014;57:2251–60. 25112376 10.1007/s00125-014-3348-z

[14] Bucay N Sarosi I Dunstan CR Osteoprotegerin-deficient mice develop early onset osteoporosis and arterial calcification. Genes Dev 1998;12:1260–8. 9573043 10.1101/gad.12.9.1260 PMC316769

[15] Albu A Bondor CI Craciun AM Circulating osteoprotegerin and asymptomatic carotid atherosclerosis in postmenopausal non-diabetic women. Adv Med Sci 2014;59:293–8. 25240503 10.1016/j.advms.2014.08.002

[16] Browner WS Lui LY Cummings SR Associations of serum osteoprotegerin levels with diabetes, stroke, bone density, fractures, and mortality in elderly women. J Clin Endocrinol Metab 2001;86:631–7. 11158021 10.1210/jcem.86.2.7192

[17] Michowitz Y Goldstein E Roth A The involvement of tumor necrosis factor-related apoptosis-inducing ligand (TRAIL) in atherosclerosis. J Am Coll Cardiol 2005;45:1018–24. 15808757 10.1016/j.jacc.2004.12.065

[18] Osmancik P Teringova E Tousek P Prognostic value of TNF-related apoptosis inducing ligand (TRAIL) in acute coronary syndrome patients. PLoS One 2013;8:e53860. 23441146 10.1371/journal.pone.0053860 PMC3575326

[19] Cheng W Liu F Wang Z Soluble TRAIL concentration in serum is elevated in people with hypercholesterolemia. PLoS One 2015;10:e0144015. 26633016 10.1371/journal.pone.0144015 PMC4669162

[20] Rochette L Meloux A Rigal E The role of osteoprotegerin in the crosstalk between vessels and bone: its potential utility as a marker of cardiometabolic diseases. Pharmacol Ther 2017;182:115–32. 28867452 10.1016/j.pharmthera.2017.08.015

[21] Makarovic S Makarovic Z Steiner R Osteoprotegerin and vascular calcification: clinical and prognostic relevance. Coll Antropol 2015;39:461–8. 26753467

[22] Toffoli B Bernardi S Candido R Osteoprotegerin induces morphological and functional alterations in mouse pancreatic islets. Mol Cell Endocrinol 2011;331:136–42. 20832449 10.1016/j.mce.2010.08.019

[23] Harper E Forde H Davenport C Vascular calcification in type 2 diabetes and cardiovascular disease: Integrative roles for OPG, RANKL and TRAIL. Vascul Pharmacol 2016;82:30–40. 26924459 10.1016/j.vph.2016.02.003

[24] Cartland SP Harith HH Genner SW Non-alcoholic fatty liver disease, vascular inflammation and insulin resistance are exacerbated by TRAIL deletion in mice. Sci Rep 2017;7:1898. 28507343 10.1038/s41598-017-01721-4 PMC5432513

[25] Clark N Peripheral arterial disease in people with diabetes. Diabetes Care 2003;26:3333. 14633825 10.2337/diacare.26.12.3333

[26] Olin JW White CJ Armstrong EJ Peripheral artery disease, evolving roles of exercise, medical therapy, and endovascular options. J Am Coll Cardiol 2016;67:1339. 10.1016/j.jacc.2015.12.049 26988957

[27] Ko SH Bandyk DF Interpretation and significance of ankle-brachial systolic pressure index. Sem Vas Sur 2013;26:86–94. 10.1053/j.semvascsurg.2014.01.002 24636605

[28] Potier L Abi Khalil C Mohammedi K Use and utility of ankle brachial index in patients with diabetes. Eur J Vasc Endovasc Surg 2011;41:110. 21095144 10.1016/j.ejvs.2010.09.020

